# Effects of Interval Walking Training on Oral Health Status in Middle-Aged and Older Adults: A Case-Control Study

**DOI:** 10.3390/ijerph192114465

**Published:** 2022-11-04

**Authors:** Tasuku Yoshimoto, Yoko Hasegawa, Mayuka Furihata, Akihiro Yoshihara, Masako Shiramizu, Ma. Therese Sta. Maria, Shoko Hori, Mayuko Morikawa, Pinta Marito, Noboru Kaneko, Kaname Nohno, Hiroshi Nose, Shizue Masuki, Takahiro Ono

**Affiliations:** 1 Division of Comprehensive Prosthodontics, Faculty of Dentistry & Graduate School of Medical and Dental Sciences, Niigata University, Niigata 951-8514, Japan; 2 Department of Sports Medical Sciences, Shinshu University Graduate School of Medicine, Matsumoto 390-8621, Japan; 3 Division of Preventive Dentistry, Faculty of Dentistry & Graduate School of Medical and Dental Science, Niigata University, Niigata 951-8514, Japan; 4 Department of Oral Health and Welfare, Faculty of Dentistry & Graduate School of Medical and Dental Sciences, Niigata University, Niigata 951-8514, Japan; 5 College of Dentistry, Manila Central University, Caloocan 1400, Philippines; 6 Faculty of Dentistry, University of Indonesia, Depok 16424, Indonesia

**Keywords:** aged, physical fitness, walking, exercise, aerobic, oral function, occlusal force

## Abstract

The purpose of this study was to determine the effect of walking training “Interval Walking Training (IWT)” on oral health status. Participants were divided into two groups: an exercise intervention group and a non-intervention group (control). The intervention group consisted of 59 subjects (20 males, 39 females) aged 50 years or older who participated in the IWT program in Matsumoto from 2019 to April 2022. The control group consisted of 33 subjects (14 males and 19 females) aged 50 years or older who have visited Niigata University Medical and Dental Hospital and agreed to participate in the study. The intervention group underwent walking training (interval walking training) for at least 5–6 months. The walking training consisted of five sets of fast walking above 70% peak aerobic capacity for walking (VO_2_peak) for 3 min, followed by 3 min of slow walking at ~40% VO_2_ peak per day for more than four days/week. The oral health status was evaluated for the number of teeth, occlusal force, salivary occult blood, masticatory performance, and tongue pressure. A total of 57 participants were analyzed in the intervention group (18 males and 39 females, age: 66.7 ± 0.8 (mean ± S.E.) years) and 33 participants in the control group (14 males and 19 females, age: 74.5 ± 1.1 (mean ± S.E.) years). There were no significant differences in gender, salivary occult blood, tongue pressure, masticatory performance, or occlusal force between the two groups at the start of the intervention (*p* = 0.36, *p* = 0.48, *p* = 0.42, *p* = 0.58, and *p* = 0.08, respectively by unpaired *t*-test or χ^2^ test). On the other hand, there were significant differences in age and BMI, with a trend toward lower age and higher BMI in the intervention group (*p* < 0.001 and *p* < 0.001, respectively, by unpaired *t*-test). In terms of rate of change, the intervention group showed a significant increase in occlusal force (F = 4.5, *p* = 0.04, ANCOVA) and a significant decrease in BMI (F = 7.3, *p* = 0.009, ANCOVA). No significant differences were observed in the other measured items. It was found that walking training in both middle-aged and older people does not only affect the physical aspect of weight loss but may help maintain and improve the occlusal force.

## 1. Introduction

Senile muscle atrophy (sarcopenia) is an age-related decrease in skeletal muscle mass [1] that is associated with an age-related decline in physical fitness, activities of daily living (ADL), and quality of life (QOL). It is considered one of the factors leading to the need for nursing care [2]. In today’s aging society, the number of older people requiring long-term care is increasing. As a result, extending healthy life expectancy has become a major issue in controlling long-term care insurance premium increases and improving long-term care insurance administration efficiency [3]. In an effort to achieve the extension of healthy life expectancy, the Japanese government has proposed a “shift to a prevention-oriented system” (frailty prevention) to ensure “support for independence,” which is the basic principle of long-term care insurance [4]. In the current situation, a program to improve musculoskeletal function in frailty prevention is one of the most important intervention methods to prevent and improve sarcopenia and extend healthy life expectancy [5,6].

Some works confirm that oral health is related to overall health and quality of life [7,8]. In recent years, attention has also focused on the decline in oral function with aging [9], and a large cohort study consisting of 2011 older people living in a community in Japan reported that the decline in oral function was a predictor of physical frailty, sarcopenia, long-term care need certification, and death [10]. Maintenance of oral function is thought to contribute to healthy life expectancy by enabling a well-balanced diet, maintaining normal motor and physiological functions, reducing the risk of developing lifestyle-related diseases such as diabetes and hypertension, and increasing their severity [10,11]. It has also been reported that the presence or absence of occlusal support and maintenance of occlusal force, which are essential for maintaining the masticatory function, contribute to proper nutritional intake, maintenance of nutritional status, and improvement of life expectancy [11,12].

Walking, more specifically, “interval walking training (IWT),” in which fast walking and normal walking are repeated for 3 min each, has been advocated since 1997 as a method for preventing skeletal muscle weakness and improving physical fitness [13]. The IWT can be performed at an intensity calibrated to the individual’s physical fitness without the need for facilities or staff guidance. So far, it has been shown to improve physical fitness [14], and lifestyle-related disease symptoms [15], among other outcomes. In addition, it has been suggested that the mechanism for it is by suppressing chronic systemic inflammation [16]. However, the relationship between systemic metabolic state and oral function and the effect of aerobic exercise training on periodontal status remains unknown.

Therefore, we hypothesized that IWT would have an effect on oral status, such as salivary occult blood, tongue pressure, masticatory performance, or occlusal force. Thus, we examined this hypothesis in a prospective case-control study.

## 2. Materials and Methods

### 2.1. Study Design and Participants

This research plan was conducted with the approval of the Shinshu University Human Subjects Review Committee (Approval No. 4822) and the Niigata University Ethics Review Committee for Research Involving Human Subjects (Approval No. 2018-0390). This study was registered in the University Hospital Medical Information Network Clinical Trial Registry (UMIN000040664). The study was performed in accordance with the STROBE guidelines.

Participants were enrolled in two groups: an intervention group that performed IWT and a control group that did not receive any exercise therapy. The participant enrollment was set from 2019 to 2021 and collected using a sequential sampling method. 

The intervention group consisted of middle-aged and older adults aged 50 years or older who were recruited for research cooperation through a public information magazine published by Matsumoto City, who expressed participation in IWT [13], and those who had established an exercise intervention using IWT [14].

The control group consisted of subjects aged 50 years or older who visited the Department of Preventive Dentistry, Niigata University Medical and Dental Hospital. It did not follow any special exercise program. All participants gave written consent after receiving oral and written explanations of the purpose and methods of the study.

Inclusion criteria for both the control and intervention groups were as follows: (1) no history of cardiac or pulmonary disease, (2) no history of smoking, (3) able to understand and answer the questionnaire appropriately, and (4) no dental procedures done such as tooth extraction, new dentures, or crown restorations during the study period [15,17].

Exclusion criteria for both the control and intervention groups were as follows: (1) participants who had difficulty walking and (2) participants with other respiratory, cardiovascular, orthopedic, or other diseases who were deemed unsuitable by the principal investigator [15].

### 2.2. Study Protocol

A period of five to six months was set between the first (Baseline) and the second evaluation. This 5 to 6-month period was determined by the IWT program and was chosen because previous studies have shown IWT to be effective during this period [13,14,15]. The second evaluation in the intervention group was conducted in either April or October. In each assessment, height, weight, and peak oxygen uptake (VO_2_peak) were measured, and physical function and oral health status were evaluated. Age, gender, current medical history, medical history, and medications were obtained by interview, height and weight were measured, and the body mass index (BMI) was calculated at each evaluation period. The effect of physical exercise in the intervention group was assessed using BMI and VO_2_peak.

#### 2.2.1. Physical Exercise Intervention Program (Interval Walking Training)

The intervention group performed walking training (IWT). On the day of the Baseline evaluation, each individual in the intervention group had their peak oxygen uptake (VO_2_peak) from walking measured at the gymnasium. The intervention group wore a portable calorimeter (JD Mate: Kissei Comtec, Matsumoto, Japan) [18] on their waist and walked for 3 min at rest, slow speed, medium speed, and fastest speed in this order, and the energy expenditure in the last minute of the fastest walk was set as the individual’s maximum physical fitness. Walking speed greater than 70% of the individual’s maximum fitness (≥70% VO_2_peak) was defined as fast walking, and VO_2_peak at <40% was defined as slow walking. The participants were instructed to consist of five sets of fast walking for 3 min followed by 3 min of slow walking per day for more than 4 days/week [13,14].

During IWT, a notification sound is emitted from the portable calorimeter every minute during fast walking. An alarm also sounds when switching between fast walking and normal walking. During the IWT intervention, participants in the intervention group went to a community center near their homes every two weeks, and the data were transferred from the terminal to the server via the Internet. The server’s e-Health Promotion System returned a trend graph of walking records [15]. Trainers and public health nurses provided individual exercise guidance based on the results of the IWT intervention.

#### 2.2.2. Oral Health Status Assessment

Intraoral examinations were performed by three dentists. All dentists were calibrated twice (2 h/day, 4 h in total) prior to the evaluation, and the evaluations were reconciled. To avoid measurement bias, the dentists who performed the evaluations were blinded to the purpose of the study. Whole assessments were conducted before meals and at least one hour after oral cleaning.

The examination items included the number of functional teeth, use of removable dentures, salivary occult blood test, tongue pressure, masticatory performance, and occlusal force. If the participants wore dentures daily, the oral function assessment was performed while wearing dentures [17].

##### Number of Teeth

The total number of functional teeth included the natural teeth, third molars, and treated teeth with crowns, as well as pontics of bridges and implants [17].

##### Periodontal Health Status

**Figure 1:**
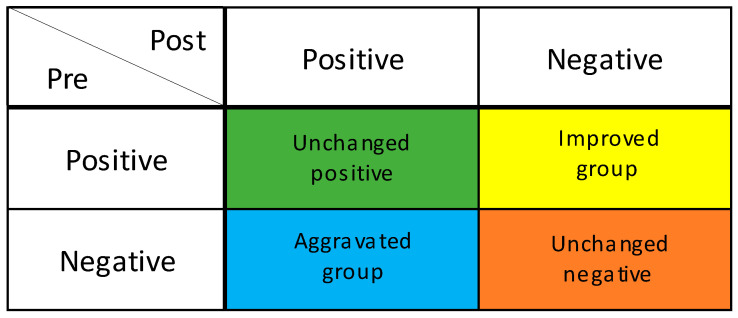
Degree of improvement in periodontal condition. GUM bleeding test; Improved group: Positive to Negative; Unchanged positive group: Positive to Positive; Unchanged negative group: Negative to Negative. Aggravated group: Negative to Positive.

The salivary occult blood test evaluates the presence of oral bleeding and is used for periodontal disease screening [19]. Participants were given 3 mL of sterile distilled water, allowed to rinse for 10 s, and then asked to spit into a paper cup to collect the specimen. The bottom edge of a hemoglobin detection test paper (Perioscreen: SUNSTAR) was dipped into the cup of the obtained specimen. When the sample solution was absorbed by the edge of the test paper, the coloration due to the reaction with the captured antibody applied in a line in the middle of the paper was observed [19]. When no coloration was observed, or the coloration was equal to the background, the test was judged as negative for periodontal disease. However, when coloration was observed in the form of lines, the test was judged as positive for periodontal disease [19]. To compare changes in periodontal disease between the intervention group and the control group, the participants were classified into four groups: the improved group, in which periodontal disease changed from positive to negative; the unchanged positive or negative group, in which positive to positive or negative to negative; and the aggravated group, in which the blood changed from negative to positive (Figure 1).

##### Tongue Pressure

Tongue pressure was measured using the JMS Tongue Pressure Monitor (TPM-02E: JMS). The balloon probe was fixed between the participant’s anterior tongue and palate, and the end of the participant’s tongue was raised toward the palate with maximum force. The balloon was pressed continuously against the palate for approximately 5 to 7 s twice, and the average value of the measurements was used as the maximum tongue pressure value of the participant [20].

##### Masticatory Performance

Masticatory performance was evaluated by the “Masticatory Performance Scoring Method” using a piece of gummy test jelly (size 20 mm × 20 mm × 10 mm, 5.5 g, UHA Mikakuto, Osaka, Japan). The participants were instructed to masticate a piece of test gummy jelly 30 times and subsequently expectorate the masticated fragments onto a piece of gauze spread over a paper cup. Any saliva adhering to the surfaces of the gauze or comminuted gummy jelly pieces was removed by running water over the surface for 30 s. The masticatory performance was evaluated by visual scoring with a 10-stage scale based on the comminuted particles of the gummy jelly [21].

##### Occlusal Force

Occlusal force was measured using a pressure-sensitive film (Dental Prescale II: G.C., Tokyo, Japan). The film was placed between the upper and lower dentition, and the participants were asked to bite with as much force as possible for about 3 s. Then the film was analyzed using an occlusal force analyzing software (Bite Force Analyzer: G.C.) to obtain the bite force measurement [22].

### 2.3. Statistical Analysis

The normality of the data distribution was verified using the Shapiro-Wilk test. When data were non-normally distributed, square root or logarithmic transformations were performed.

The sample size was calculated based on the data from previous studies that assessed the relationship between Physical Fitness and Blood Pressure in Middle-Aged and Older People [14]. The sample size was determined assuming that there was a difference in the BMI (typical physical measurement item) of the intervention group before and after the intervention. As a result, the minimum sample size was 40 (Effect size = 0.59, αerror = 0.05, power = 0.88, *t*-test without correspondence). The control group consisted of 33 subjects so that distribution of the sample mean was normally distributed with the anticipation of a 10% dropout.

For all continuous variables, the percent change from the baseline to the second evaluation was calculated.

Rate of change (%) = {(second-evaluation value − baseline value)/baseline value} × 100

To compare the intervention and control groups at baseline, we also used the unpaired *t*-test or the χ^2^ test. An analysis of covariance (ANCOVA) was used to compare the intervention and control groups in terms of rate of change in tongue pressure, masticatory performance, occlusal force, and BMI, with the main effects being between-group difference and time, and age as a covariate. The χ^2^ test was used to compare the improvement in periodontal status between the intervention and control groups.

To examine in detail the relationship between the effect of IWT and oral function in the intervention group, we examined the relationship between the effect of physical exercise (increase or decrease in VO_2_peak and BMI) and the degree of improvement in oral function and periodontal status using IWT with the unpaired *t*-test or the χ^2^ test.

SPSS (IBM, Statistics 23, Armonk, NY, USA) was used for statistical analysis, and all significance levels were set at 5%.

## 3. Results

**Figure 2:**
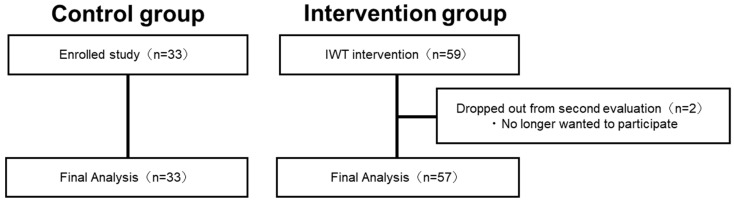
Flowchart showing the process of determining the final analysis participants.

Figure 2 shows the process of participants in the intervention group. In the intervention group, out of the 59 participants (20 males, 39 females) who participated in the pre-intervention assessment (baseline), two did not participate in the re-assessment, while 57 participants completed the second evaluation. Finally, the intervention group consisted of 57 participants (18 males and 39 females, mean age: 66.7 ± 0.8 (mean ± S.E.) years) (Figure 2). The control group consisted of 33 participants (14 males and 19 females with a mean age of 74.5 ± 1.1 (mean ± S.E.) years), and no participants dropped out. Significant effects of physical exercise were observed in the intervention group (VO_2_peak: *p* = 0.03, BMI: *p* < 0.001, paired *t*-test).

In the intervention group, out of the 59 participants who participated in the preintervention assessment, two did not participate in the re-assessment, while 57 participants completed the second evaluation. The control group consisted of 33 participants, and no participants dropped out.

Table 1 shows a summary of the participants. No significant differences were found in gender, periodontal status, tongue pressure, masticatory performance, or occlusal force between the two groups at the start of the intervention. On the other hand, there were significant differences in age and BMI, with the intervention group tending to be younger and having higher BMI.

**Figure 3:**
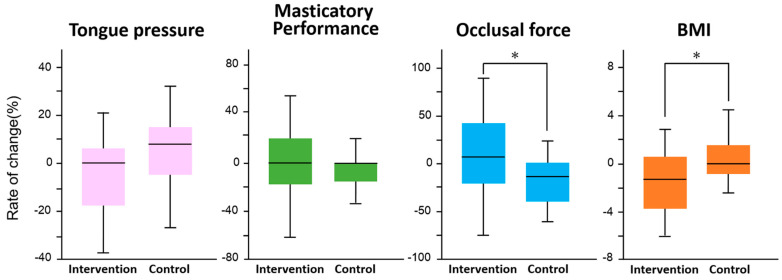
Comparison of rate of change between interval walking training and control groups in each measured item. An analysis of covariance (ANCOVA) was used to compare the intervention and control groups in terms of rate of change in tongue pressure, masticatory performance, occlusal force, and BMI, with the main effects being between-group difference and time, and age as a covariate. *: Significant difference in each measured item by ANCOVA.

**Figure 4:**
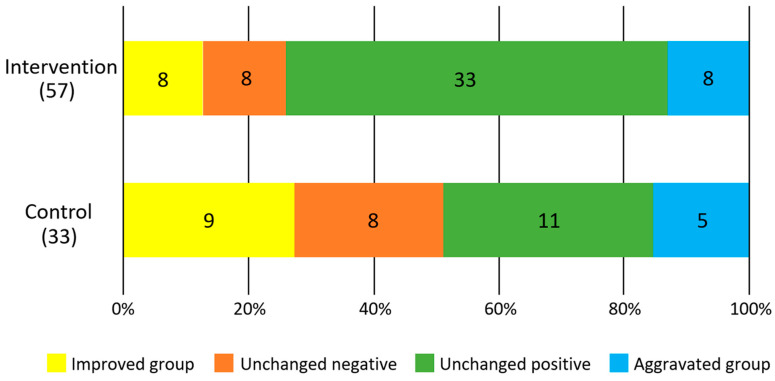
Degree of improvement in salivary blood state. Numbers in the figures show participants.

Figure 3 shows the results of the difference in the rate of change between the intervention and control groups for each survey item. In the intervention group, there was a significant increase in occlusal force (F = 5.4, *p* = 0.02) and a significant decrease in BMI (F = 6.9, *p* = 0.01). No significant changes were observed in tongue pressure (F = 2.9, *p* = 0.09) and masticatory performance (F = 0.3, *p* = 0.6). There was no significant difference in the improvement of periodontal status between the intervention and control groups (*p* = 0.33, χ^2^ test, Figure 4).

Table 2 shows the relationship between the effect of exercise, degree of improvement of gingival bleeding, and the rate of change in oral function in the intervention group. Although there was no significant relationship between the effect of exercise and the degree of improvement of gingival bleeding and the rate of change in oral function, there was a tendency for gingival bleeding to improve as BMI decreased (*p* = 0.06, χ^2^ test).

## 4. Discussion

Our results showed that IWT significantly improved the values of occlusal force and BMI. Previous studies have shown that IWT improves lifestyle-related disease indices [15], and the fact that the intervention group in this study showed improvement in BMI indicates that the IWT intervention was appropriately implemented and that there was an actual physical effect on the participants. In the participants of this study, there was no difference in oral status between the intervention and control groups at baseline, suggesting that the intervention group did not have a high occlusal force to begin with, and that the effect of IWT did not reduce the occlusal force.

Since the second evaluation in the intervention group was conducted in either April or October, we examined the effect of the difference in the evaluation period on the measured values. The results showed no significant difference in the measured values of any assessment items between April and October.

The relationship between occlusal force and physical function has been reported in several previous studies, and reports on older adults have shown that occlusal force is related to other objective physical abilities such as walking speed, muscle strength, balance function, and falls [23,24,25,26]. Matake et al. examined whether there were differences in occlusal force depending on the presence or absence and frequency of physical exercise experience in 207 healthy adults and found that the group with more physical exercise experience showed significantly higher occlusal force than the others, indicating that physical exercise experience clearly influences differences in occlusal force [27].

The occlusal force is mainly generated by the activity of masticatory muscles such as the masseter and temporalis muscles. Furthermore, it has been reported that biting increases the muscle activity of the sternocleidomastoid and trapezius muscles, which are involved in head retention [28]. The action potential of the masseter and temporalis muscles changes along with the changes in the head position [29], indicating that the masticatory muscles, together with the cervical and shoulder muscle groups, contribute to head stabilization as antigravity muscles [30]. These findings suggest that occlusal force is an evaluation that reflects the support and fixation of the lower limbs to maintain an upright posture and the stability of the head. Sasaki et al. investigated the effects of biting on both isometric and isokinetic muscle strength of the lower limbs in healthy adult males with individual normal bite and reported that there was a difference between isometric and isokinetic muscle strength in terms of the effects of biting and that effect of biting was significantly greater on isometric muscle strength than isokinetic muscle strength [31,32]. Based on the previously mentioned studies, it is thought that the physical exercise intervention by IWT led to the improvement of occlusal force because muscle activity by appropriate exercise intensity stimulates the masticatory muscles as cooperative muscles for its performance.

In this study, salivary occult blood tests were also evaluated. Because exercise intervention with IWT has been reported to improve lifestyle-related disease symptoms [15], and because exercise increases blood flow, which in turn increases gingival blood flow secondarily, we thought that periodontal status might be improved in the long term [33]. Results showed no significant differences between the intervention and control groups, but a trend toward improved gingival bleeding with decreasing BMI was observed in the intervention group. This may be due to the possible effect of IWT on periodontal status, which would be caused by the suppression of chronic inflammation [16,34,35]. On the other hand, one of the most important pathogens of periodontal disease, *Porphyromonas gingivalis*, invades blood vessels from the endothelial cells of the periodontal pocket [36].

Furthermore, its components, lipopolysaccharide (LPS, an endotoxin) and inflammatory cytokines, have been reported to adversely affect skeletal muscle regeneration and inhibit muscle repair after hard training [37,38]. This suggests that, in addition to training with IWT, periodic dental visits to control oral bacteria related to periodontal disease are necessary to obtain more effective exercise results.

It has been reported that those with impaired oral function tend to avoid foods that are difficult to chew and are more likely to suffer from poor nutrition [11]. It has also been reported that opportunities for exercise decrease as oral function declines [39]. Both undernutrition and lack of exercise are causes of physical function decline and loss of muscle mass, and early detection and intervention are considered the keys to improving prognosis [40].

The improvement of oral functions by IWT revealed in this study indicates that the effect of IWT is not only a simple improvement of motor function but may secondarily help maintain and improve the occlusal force. In addition, since no clear intervention effects could be confirmed for oral functions other than occlusal force, it could be inferred that the changes to the oral cavity brought about by physical exercise are localized.

There are several limitations to this study. The control group was limited to those who regularly visited preventive dentistry, so it is possible that there was a difference in oral health between the intervention and control groups because many of them were highly conscious of their oral health. In addition, systemic diseases and medication status were not considered in this study.

## 5. Conclusions

The results of this study indicate that sustained exercise training in middle-aged and older adults does not only affect the physical aspect of weight loss but also affects the occlusal force of oral function.

## Figures and Tables

**Table 1:** Participant characteristics of each group before intervention.

		Intervention (n = 57)	Control (n = 33)	*p*
Age(years) *		66.7 ± 0.8	74.5 ± 1.1	<0.001
Gender	Male (%)	18(31.6)	14(42.4)	0.36
	Female (%)	39(68.4)	19(57.6)	
Number of teeth		25.3 ± 0.7	25.2 ± 0.6	0.37
Gum bleeding	Positive (%)	18(72)	15(45.5)	0.48
	Negative (%)	7(28)	18(54.5)	
Tongue pressure(kPa)		36.2 ± 1.0	34.4 ± 0.9	0.42
Masticatory Performance (Score)		5.6 ± 0.3	5.9 ± 0.3	0.58
Occlusal force(N)		379.4 ± 27.0	468.9 ± 41.9	0.08
BMI *		24.0 ± 0.4	21.4 ± 0.5	<0.001

Data are presented as the mean ± standard error or number of participants (%). Intervention: Interval walking training group. Control: Non-training group. Number of functional teeth: Natural teeth and treated teeth that have a crown, as well as bridge pontics and implants. Gum bleeding: Periodontal condition assessment by detection of hemoglobin in the obtained mouth rinse solution. Oral dryness: Oral dryness evaluation by an oral moisture meter (Mucus^®^: Panasonic). Tongue pressure: Maximum tongue pressure evaluation by the JMS Tongue Pressure Meter (TPM-02E: JMS). Masticatory performance (Score): Gummy Jelly Score evaluation by visual inspection. Occlusal force: Evaluation of Biting Force by Pressure-Sensitive Film (Dental Prescale II: G.C., Tokyo) for Occlusal Force Measurement System. BMI (Body Mass Index): A measurement of someone’s weight in relation to their height. *p:* Two-Group Comparison with the unpaired t-test or the χ^2^ test. *: Significant difference in each measured item by the unpaired *t*-test or the χ^2^ test.

**Table 2:** Relationship between the effect of exercise and the improvement of gingival bleeding or the rate of change in oral function in the intervention group.

		Change in BMI		*p*	*Change* in VO_2_peak		*p*
		Decrease	Increase		Decrease	Increase	
GUM bleeding	Improved group (%)	7(17.9)	3(16.7)	0.06	1(6.3)	7(21.2)	0.47
	Unchanged negative (%)	7(17.9)	0(0)		2(12.5)	4(12.1)	
	Unchanged positive (%)	22(56.5)	11(61.1)		10(62.5)	19(57.6)	
	Aggravated group (%)	3(7.7)	4(22.2)		3(18.7)	3(9.1)	
Rate of change in Tongue pressure		−1.2 ± 3.3	5.3 ± 6.6	0.32	−1.2 ± 5.1	0.2 ± 4.3	0.86
Rate of change in Masticatory Performance		16.7 ± 10.2	5.5 ± 8.5	0.47	24.7 ± 15.8	10.0 ± 9.9	0.42
Rate of change in Occlusal force		12.6 ± 8.7	6.0 ± 10.3	0.68	6.9 ± 17.3	6.8 ± 8.2	0.99

Data are presented as the mean ± standard error or number of participants (%). Change in BMI: Second evaluation BMI value-baseline BMI value. Change in VO_2_peak: Second evaluation VO_2_peak value-baseline VO_2_peak value. *p:* Two-Group Comparison with the unpaired t-test or the χ^2^ test. Variables are the same as in Table 1.

## Data Availability

The materials described in the manuscript, including all relevant raw data, will be freely available to any scientist wishing to use them for non-commercial purposes by contacting the corresponding author without breaching patient confidentiality.
